# Heat-Killed *Saccharomyces boulardii* Alleviates Dextran Sulfate Sodium-Induced Ulcerative Colitis by Restoring the Intestinal Barrier, Reducing Inflammation, and Modulating the Gut Microbiota

**DOI:** 10.3390/nu16050702

**Published:** 2024-02-29

**Authors:** Yuxin Jin, Jingwei Wu, Kunlun Huang, Zhihong Liang

**Affiliations:** 1 College of Food Science and Nutritional Engineering, China Agricultural University, Beijing 100083, China; j17860722880@163.com (Y.J.); wjwww1123@163.com (J.W.); hkl009@163.com (K.H.); 2 Beijing Laboratory for Food Quality and Safety, College of Food Science and Nutritional Engineering, China Agricultural University, Beijing 100083, China

**Keywords:** *Saccharomyces boulardii*, probiotic, heat-killed, postbiotics, β-glucan, prebiotics, ulcerative colitis, gut microbiota

## Abstract

Ulcerative colitis (UC) is a global intestinal disease, and conventional therapeutic drugs often fail to meet the needs of patients. There is an urgent need to find efficient and affordable novel biological therapies. *Saccharomyces boulardii* has been widely used in food and pharmaceutical research due to its anti-inflammatory properties and gut health benefits. However, there is still a relatively limited comparison and evaluation of different forms of *S. boulardii* treatment for UC. This study aimed to compare the therapeutic effects of *S. boulardii*, heat-killed *S. boulardii*, and *S. boulardii* β-glucan on UC, to explore the potential of heat-killed *S. boulardii* as a new biological therapy. The results demonstrate that all three treatments were able to restore body weight, reduce the disease activity index (DAI), inhibit splenomegaly, shorten colon length, and alleviate histopathological damage to colonic epithelial tissues in DSS-induced colitis mice. The oral administration of *S. boulardii*, heat-killed *S. boulardii*, and *S. boulardii* β-glucan also increased the levels of tight junction proteins (Occludin and ZO-1), decreased the levels of pro-inflammatory cytokines (TNF-α, IL-1β, and IL-6) in the serum, and suppressed the expressions of TNF-α, IL-1β, and IL-6 mRNA in the colon. In particular, in terms of gut microbiota, *S. boulardii*, heat-killed *S. boulardii*, and *S. boulardii* β-glucan exhibited varying degrees of modulation on DSS-induced dysbiosis. Among them, heat-killed *S. boulardii* maximally restored the composition, structure, and functionality of the intestinal microbiota to normal levels. In conclusion, heat-killed *S. boulardii* showed greater advantages over *S. boulardii* and *S. boulardii* β-glucan in the treatment of intestinal diseases, and it holds promise as an effective novel biological therapy for UC. This study is of great importance in improving the quality of life for UC patients and reducing the burden of the disease.

## 1. Introduction

Inflammatory bowel disease (IBD) is a type of autoimmune disease characterized by chronic inflammation of the intestinal mucosa. IBD mainly includes ulcerative colitis (UC) and Crohn’s disease (CD), with UC being the most common form. Western, developed countries are the regions with the highest incidence and prevalence of UC worldwide, particularly in Europe, where UC is recognized as a common gastrointestinal disorder [1]. According to statistics, the incidence of UC in Europe is as high as 24 per 100,000 individuals, with prevalence ranging from 2.4 to 294 per 100,000 individuals [2]. The United States has the highest prevalence of UC, and statistics show that approximately USD 1 to 1.5 billion are spent annually on the treatment of UC [3]. With urbanization and the modernization of daily life, the incidence and prevalence of UC continue to rise, posing significant threats to people’s quality of life and health [4]. Currently, the treatment methods for UC suffer from issues such as high cost, short duration of symptom relief, and frequent adverse reactions [5]. There is an urgent need for new preventive and therapeutic approaches to improve disease management.

UC presents as inflammation of the rectal and colonic mucosa, with a specific site of onset. However, the pathogenesis of UC is complex and influenced by multiple factors, making it difficult to provide targeted treatment [6]. Research into the interplay between the gut microbiota and the intestinal mucosal immune system, resulting in inflammation, is progressively increasing [7]. The targeted modulation of the gut microbiota may alleviate symptoms associated with UC and provide a new strategy for its treatment. Therefore, it is of great significance to rationally select probiotics, prebiotics, or postbiotics for future applications.

*Saccharomyces boulardii* (*S. boulardii*), a subspecies of Saccharomyces cerevisiae (*S. cerevisiae*), is a probiotic yeast. With the increasing demand for functional foods containing probiotics, *S. boulardii* has been widely used in the production and processing of food [8]. *S. boulardii* also exhibits remarkable potential in the treatment of UC due to its immunomodulatory, anti-inflammatory, and anticancer properties in the body [9,10]. However, previous studies have indicated that factors such as processing and storage conditions (such as time, temperature, and water activity) in industrial production can affect the viability of probiotics, thereby reducing the viable count and colonization efficiency in the gut [11]. Recent research has suggested that the beneficial effects of probiotics may not necessarily be directly related to live bacteria, but rather to the metabolic byproducts and cellular components of the bacteria, which could be key factors in promoting health [12]. Therefore, postbiotics have gradually gained attention and become a new research direction as potential alternatives to probiotics [13,14]. The efficacy of postbiotics is based on the complex molecules generated by microbes, including proteins, lipids, carbohydrates, cell wall components, and fermentation products. However, research on the functions of various forms of postbiotics is still in its early stages and requires further studies to enrich our understanding of their effects and mechanisms. β-glucan in the cell wall of *S. boulardii* (referred to as *S. boulardii* β-glucan) is a natural polysaccharide considered a typical prebiotic. Although studies have shown that S. boulardii β-glucan exhibits a certain efficacy in the treatment of intestinal inflammation [15,16,17], there are few studies directly comparing the therapeutic effects of prebiotics, postbiotics, and probiotics.

Combining the research hotspot of postbiotics, this work aimed to clarify the effects of heat-killed *S. boulardii* on the improvement of DSS-induced colitis in mice and to make a preliminary comparison of the effectiveness of probiotic (*S. boulardii*), postbiotic (heat-killed *S. boulardii*), and prebiotic (*S. boulardii* β-glucan) interventions. This study also investigated the potential mechanisms of UC model pathogenesis and treatment from the perspectives of the intestinal barrier, inflammatory response, and gut microbiota.

## 2. Materials and Methods

### 2.1. Chemicals and Reagents

Fubon feed additive and *S. boulardii* β-glucan (purity 80%, molecular mass 800–1000 kDa) were purchased from Angel Yeast Co., Ltd. (Yichang, China). Yeast Extract Peptone Dextrose Medium (YPD) was purchased from Qingdao Hi-Tech Park Haibo Biotechnology. Dextran sulfate sodium (DSS, *w*/*v*. molecular mass 36–50 kDa) was purchased from Yisheng Biotechnology Co., Ltd. (Shanghai, China). RIPA buffer (high) and PMSF were purchased from Solarbio Science & Technology Co., Ltd. (Beijing, China). Protease inhibitor, ZO-1 Rabbit Polyclonal Antibody, Occludin Rabbit Polyclonal Antibody, GAPDH Rabbit Polyclonal Antibody, Horseradish peroxidase-conjugated goat anti-rabbit IgG(H + L) secondary antibody, and an ELISA Kit (Mouse TNF-α, IL-1β, and IL-6) were purchased from Beyotime Biotechnology Co., Ltd. (Shanghai, China). TransZolTM Up Plus RNA Kit and Reverse transcriptase reagent kit were purchased from Beijing TransGen Biotechnology Co., Ltd. (Beijing, China). SuperReal PreMix Plus (SYBR Green) was purchased from Tiangen Biochemical Technology Co., Ltd. (Beijing, China).

### 2.2. Animals

Male specific pathogen-free (SPF) C57BL/6J mice (age, 6–8 weeks; weight, 18–22 g) were purchased from Beijing Vital River Laboratory Animal Technology Co., Ltd. (SCXK (Beijing, China) 2021-0006). All mice were housed in a standard SPF environment in the animal center (SYXK (Jing) 2020-0052). Four mice were maintained in an individually ventilated cage (temperature, 22 ± 2 °C; relatively humidity, 40–70%; standard 12 h/12 h light/dark cycle). The study was conducted in accordance with the Guidelines for the Care and Use of Laboratory Animals at China Agricultural University and was approved by the Animal Ethics Committee of the Ministry of Agriculture’s Genetically Modified Organisms Testing Center (approval number: Aw01503202-4-4; approval date: 7 April 2023).

### 2.3. Preparation of Probiotics and Postbiotics

*S. boulardii* was isolated from Angel Fubon feed additive. Based on morphological and genetic sequence comparisons, we identified the isolated strain as *S. boulardii* (Supplementary Figure S1), with the strain’s preservation number being CCTCC NO.M 2012116.

The *S. boulardii* strain was inoculated in a YPD liquid medium and cultured at 30 °C with shaking at 200 rpm until reaching the stationary phase (7 × 10^8^ CFU/mL). The sterile fermented liquid was transferred to a 50 mL centrifuge tube and subjected to heat treatment in a 100 °C water bath for 20 min. The heat-killed *S. boulardii* cells were obtained via centrifugation (6000 rpm for 10 min at 4 °C) and washed three times with sterile phosphate-buffered saline (PBS). After pre-freezing was completed, the cells were freeze-dried using a freeze dryer, and a lyophilized postbiotic sample was prepared. Finally, the freeze-dried sample was gently grinded to obtain 600 mg of postbiotic powder.

### 2.4. Animal Experiment Design

**Figure 1:**
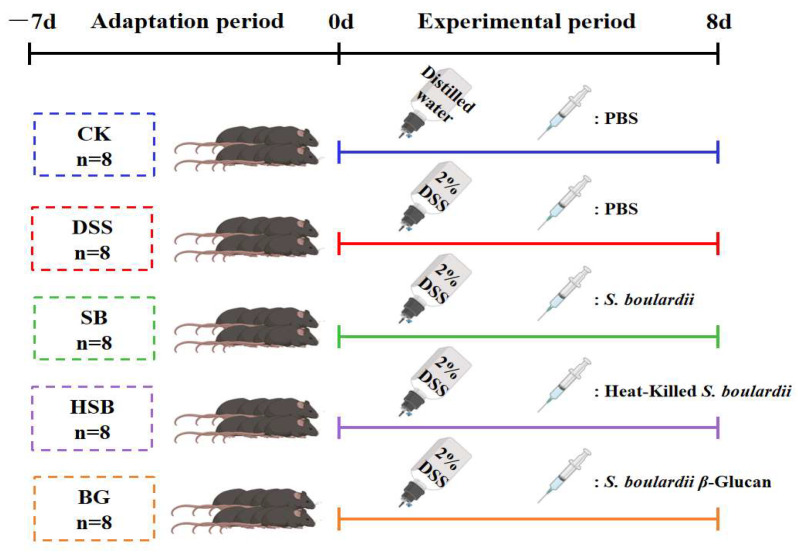
Animal experiment design.

After forty C57BL/6J male mice were acclimatized to and fed in the experimental environment for a week, the mice were divided into five groups (*n* = 8): CK (Blank control), DSS, SB (*S. boulardii*), HSB (heat-killed *S. boulardii*), and BG (*S. boulardii* β-glucan). Mice in the DSS, SB, HSB, and BG groups were administered with DSS (2%) in drinking water daily for 8 days. The 2% DSS solution was changed once a day for 8 days. The mice were weighed daily, and their disease activity index (DAI) was assessed according to the scoring criteria shown in Supplementary Table S1. Meanwhile, mice in the SB, HSB, and BG groups were gavaged with 200 μL of *S. boulardii* (10^7^ CFU/0.2 mL), heat-killed *S. boulardii* (10^7^ CFU/0.6 mg/0.2 mL), and *S. boulardii* β-glucan (0.6 mg/0.2 mL) for 8 days. Mice in the CK and DSS groups were gavaged with 200 μL of sterile phosphate-buffered saline (PBS) solution. Both postbiotics and prebiotics were dissolved in the PBS solution (Figure 1). On the 16th day, whole blood samples were obtained from the mice via ocular blood collection and left at room temperature for 2 h. Subsequently, the blood samples were centrifuged at 4 °C 3500 rpm for 15 min to obtain serum. The collected serum was stored at −80 °C until further analysis. Following the blood collection, all mice were euthanized via cervical dislocation, and immediate dissections were performed. The liver, kidneys, and spleen were weighed to calculate the organ index. The organ index (the spleen, liver, and kidney organ index) was calculated as organ weight/body weight × 100%. The colon was isolated, and the length of the colon was measured. Then, the distal colon (2 cm from the anus) was fixed with 4% paraformaldehyde. The remaining colon section and cecal contents were immediately frozen in liquid nitrogen and stored at −80 °C for further analysis.

### 2.5. Histopathological Analysis

The fixed 2 cm colon tissue was embedded in paraffin and cut into 4 µm thick sections. After dewaxing the paraffin sections, they were stained with hematoxylin and eosin. The sections were examined using a SUNNY EX20 biological microscope (Ningbo Sunny Instrument Co., Ltd., Ningbo, China).

### 2.6. Measurement of Tight Junction Protein Levels in Colon Tissue

The total proteins were extracted from the colon using RIPA buffer (high), protease inhibitor, and PMSF in a 100:1:1 ratio. At 4 °C, they were centrifuged at 12,000× *g* for 10 min, and the supernatant was collected. After determining the total protein concentration using the BCA protein quantification kit, the proteins were separated via SDS-PAGE gel electrophoresis. Then, they were transferred onto a PVDF membrane and sealed with 5% skim milk powder for 1 h. Next, they were incubated with ZO-1 Rabbit Polyclonal Antibody (1:1000) and Occludin Rabbit Polyclonal Antibody (1:1000) primary antibodies separately overnight at 4 °C. The membrane was washed 3 times with TBST, and Horseradish peroxidase-conjugated goat anti-rabbit IgG(H + L) secondary antibody (1:2000) was added, incubating on a shaker at room temperature for 1 h. The membrane was then washed 3 times with TBST. After incubating the membrane with ECL in a darkroom, the protein bands were visualized using a chemiluminescence imaging system. GAPDH served as an internal reference; the relative expression level of the target protein was determined by calculating the ratio of the intensity values between the target protein band and the internal reference band.

The total RNA was extracted from the colon using the TransZolTM Up Plus RNA Kit and then reverse transcribed into complementary DNA (cDNA) using a reverse transcriptase reagent kit. The cDNA was subjected to quantitative real-time polymerase chain reaction (RT-qPCR) analysis using SuperReal PreMix Plus (SYBR Green). Finally, the 2^−ΔΔCt^ method was used to determine the mRNA expression levels of Occludin and ZO-1 in the colon tissue relative to the expression level of GAPDH. The primer sequences used in this study are listed in Supplementary Table S2.

### 2.7. Measurement of Pro-Inflammatory Cytokine Levels in Serum and Colon Tissue

ELISA kits were used according to the manufacturer’s instructions to measure the levels of pro-inflammatory cytokines TNF-α, IL-1β, and IL-6 in serum.

The total RNA was extracted from the colon using the TransZolTM Up Plus RNA Kit, and then reverse transcribed into complementary DNA (cDNA) using a reverse transcriptase reagent kit. The cDNA was subjected to quantitative real-time polymerase chain reaction (RT-qPCR) analysis using SuperReal PreMix Plus (SYBR Green). Finally, the 2^−ΔΔCt^ method was used to determine the mRNA expression levels of TNF-α, IL-1β, and IL-6 in the colon tissue relative to the expression level of GAPDH. The primer sequences used in this study are listed in Supplementary Table S2.

### 2.8. Gut Microbiota Analysis

The total DNA of the microorganisms was extracted from fecal samples using QIAamp DNA isolation Kits (QIAamp, Hilden, Germany). The 16S rRNA gene V3–V4 region sequences of the samples were amplified using the primers 338F (5′-ACTCCTACGGGAGGCAGCAG-3′) and 806R (5′-GGACTACHVGGGTWTCTAAT-3′). Subsequently, sequencing was performed on the Nova-Seq platform (Illumina, San Diego, CA, USA). The raw data were filtered and analyzed using QIIME (v1.9.1). The OTUs at a similarity threshold of 97% were clustered using UPARSE (v7.0.1001). The determination of the alpha and beta diversities was also performed in QIIME (v1.9.1). PCoA and NMDS plots were generated on the Majorbio Cloud Platform based on the weighted-unifrac distance algorithm. Heatmaps were created using HemI software (v1.0.3.7). In following linear discriminant analysis (LDA), an LDA effect size (LEfSe) analysis was conducted to identify differentially abundant bacterial taxa from the phylum to the species level using two filters (*p* < 0.05 and LDA score > 4). PICRUSt was utilized to predict the functional features of microbial communities, while STAMP (version 2.1.3) was used for visualizing significant statistical differences in functional features among different samples. The analysis platform was provided by Novogene Co., Ltd. (Beijing, China) (https://magic.novogene.com, accessed on 20 December 2023).

### 2.9. Statistical Analysis

All data were expressed as means ± SEM and analyzed using the GraphPad Prism 9.0 program (GraphPad Software, San Diego, CA, USA). All data differences were analyzed via one-way analysis of variance (ANOVA), followed by *Tukey’s* test, and *p* < 0.05 was considered statistically significant.

## 3. Results

### 3.1. Heat-Killed S. boulardii Alleviated the Symptoms of Colitis

**Figure 2:**
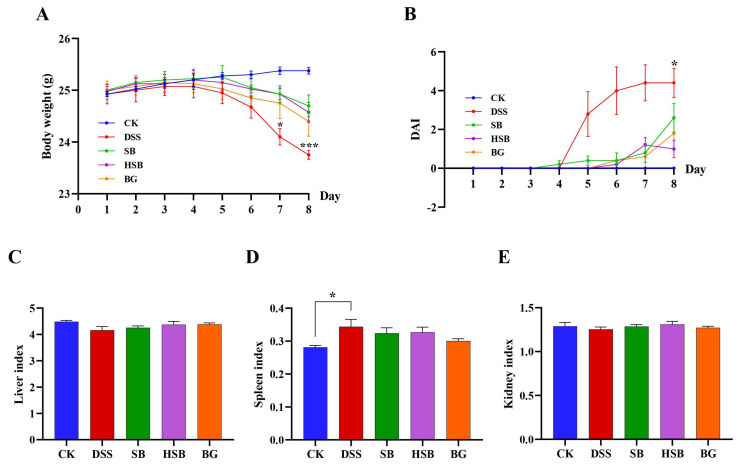
Effects of heat-killed *S. boulardii* on the body weight curve and the organ indices of mice with DSS-induced colitis. (**A**) Body weight curve. (**B**) Disease activity index (DAI). (**C**) Liver index. (**D**) Spleen index. (**E**) Kidney index. Note: Data are expressed as mean ± SEM. A comparison of significant differences between groups is indicated, where * *p* < 0.05 and *** *p* < 0.001 compared with the CK group.

By constructing a DSS-induced colitis model and administering *S. boulardii*, heat-killed *S. boulardii*, and *S. boulardii* β-glucan orally for 8 consecutive days, we could visually compare the therapeutic effects of these three treatments on mice UC symptoms. The body weight change curve shows that, except for the control group, the body weight of animals in the remaining groups started to decrease on the 5th day. Compared to the control group, the DSS group showed a significant decrease in body weight on the 7th day (*p* < 0.05), while the SB, HSB, and BG groups exhibited a significantly slower decline in body weight, indicating that all three treatments can delay the decline in body weight in UC mice (Figure 2A). The disease activity index (DAI) was used to reflect the disease status and severity of the animals in each group. The DAI in the DSS group showed a significant increase starting from the 5th day and displayed a significant difference compared to the control group upon induction on the 8th day. Although the SB, HSB, and BG groups also had an increase in DAI, there were no significant differences compared to the control group, indicating that the colitis symptoms induced by DSS in mice were partially alleviated to some extent (Figure 2B).

Furthermore, by evaluating the liver, spleen, and kidney indices, we could gain some understanding of the biological functions of vital organs. Specifically, the spleen, as an important peripheral immune response organ, may become enlarged due to immune activation during DSS-induced colitis. The results show that compared to the control group, only the mice in the DSS group showed a significant increase in the spleen index (Figure 2D, *p* < 0.05), while there were no significant differences in the spleen index between each treatment group and control group (*p >* 0.05). However, there were no significant differences observed in the liver and kidney indices among the groups (Figure 2C–E).

In summary, we can see that heat-killed *S. boulardii* can effectively alleviate the symptoms of DSS-induced colitis. The assessment in terms of animal weight, DAI index, and organ index showed similar therapeutic effects compared to the *S. boulardii* and *S. boulardii* β-glucan treatment groups.

### 3.2. Heat-Killed S. boulardii Alleviated Colonic Shortening and Pathological Changes

**Figure 3:**
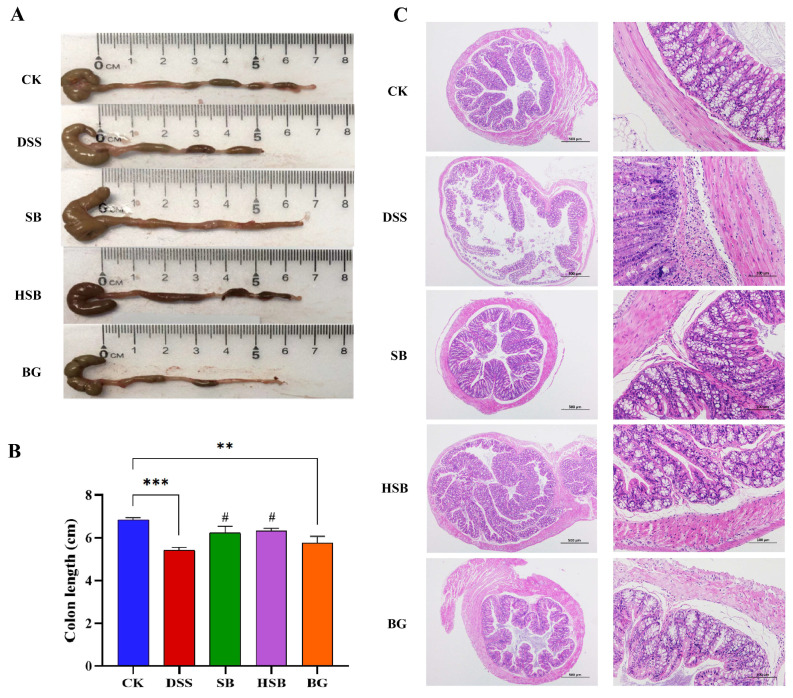
Effects of heat-killed *S. boulardii* on colonic length and pathological changes in DSS-induced mice. (**A**) Apparent picture of colon tissue. (**B**) Colon length. (**C**) Hematoxylin–eosin staining result of colon tissue. (at 40× and 200× magnification) at day 8. Note: Data are expressed as mean ± SEM. A comparison of significant differences between groups is indicated, where ** *p* < 0.01 and *** *p* < 0.001 compared with the CK group; # *p* < 0.05 compared with the DSS group.

Compared to the control group, mice in the DSS group showed a significant reduction in colon length (*p* < 0.001). Although the extent of colon shortening in the BG group was less than that in the DSS group, there was still a significant difference compared to the control group (*p* < 0.01). In contrast, mice in both the SB and HSB groups had significantly longer colon lengths compared to the DSS group (*p* < 0.05) and showed no significant difference compared to the control group (*p* > 0.05) (Figure 3A,B).

Furthermore, a histopathological analysis revealed that the colon tissue structure in the control group mice was normal, with intact colonic mucosa, regular glandular arrangement, and no inflammatory cell infiltration. In contrast, mice in the DSS group exhibited colon mucosal damage, deformation, and the detachment of intestinal villi. At a magnification of 200×, it was observed that the crypt structures in the colon were abnormal, with irregular or even absent glandular arrangements, as well as the infiltration of inflammatory cells. Compared to the DSS group, the mice in the SB, HSB, and BG groups all showed varying degrees of the reversal of colon injury, characterized by mostly intact mucosa, relatively preserved glands and crypts, and mild inflammation, approaching the characteristics of normal colon tissue (Figure 3C). It can be seen that *S. boulardii*, heat-killed *S. boulardii*, and *S. boulardii* β-glucan can all, to varying degrees, restore colon shortening and damage in DSS-induced colitis mice. Among them, *S. boulardii* and heat-killed *S. boulardii* showed significantly better recovery effects compared to *S. boulardii* β-glucan.

### 3.3. Heat-Killed S. boulardii Increased the Content of Colonic Tight Junction Proteins

**Figure 4:**
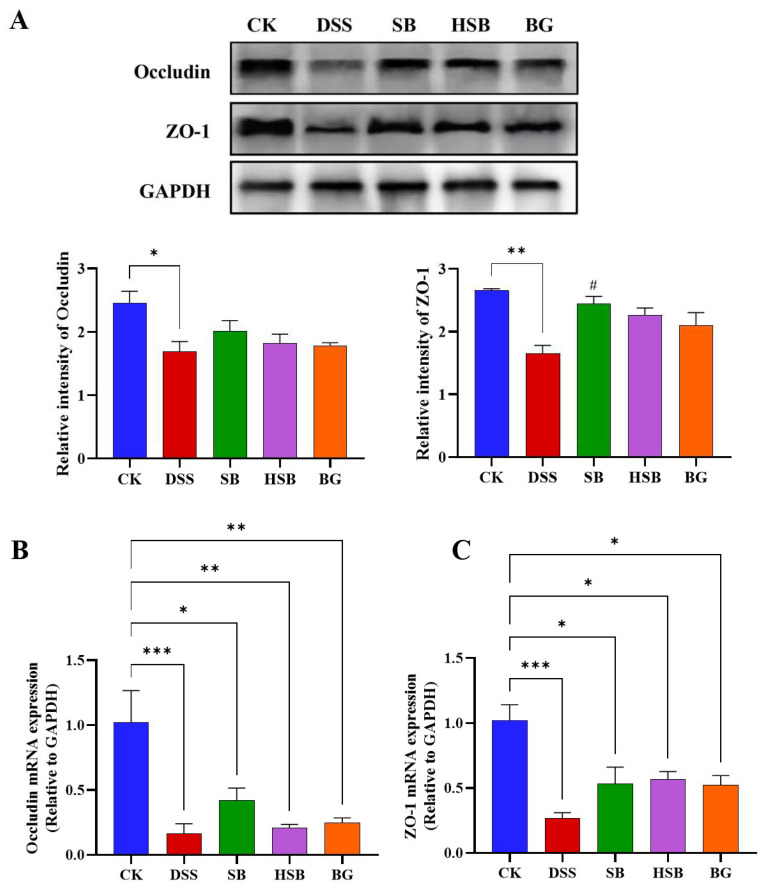
Effect of heat-killed *S. boulardii* on the levels of tight junction proteins in the colon of DSS-induced mice. (**A**) The relative expression levels of the proteins Occludin and ZO-1. (**B**) The mRNA expression level of Occludin. (**C**) The mRNA expression level of ZO-1. Note: Data are expressed as mean ± SEM. A comparison of significant differences between groups is indicated, where * *p* < 0.05, ** *p* < 0.01, and *** *p* < 0.001 compared with the CK group; # *p* < 0.05 compared with the DSS group.

To comprehensively investigate the extent of intestinal barrier damage in DSS-induced colitis mice and the effects of heat-killed *S. boulardii* on the intestinal barrier, we used Western blots to measure the levels of tight junction proteins Occludin and ZO-1 in the colon. Compared to the control group, the levels of Occludin (*p* < 0.05) and ZO-1 (*p* < 0.01) were significantly reduced in the colon of the DSS-induced colitis group (Figure 4A). Compared to the DSS group, all treatment groups showed a recovery in the levels of tight junction proteins, with the SB group demonstrating the most significant effect by significantly increasing the levels of ZO-1 in the colon (*p* < 0.05). Overall, the impact of heat-killed *S. boulardii* on the content of colonic tight junction proteins was not as significant as live that of *S. boulardii*, but it had a similar effect to that of *S. boulardii β*-glucan. This result indicates that heat-killed *S. boulardii* can partially alleviate DSS-induced intestinal barrier damage.

By detecting the expression levels of tight junction proteins Occludin and ZO-1 mRNA in the colon, we can understand how heat-killed *S. boulardii* affects the regulation of tight junction proteins in colitis mice. We used the RT-qPCR technique to measure the levels of Occludin and ZO-1 in the colons of different groups of mice. Compared to the control group, the expression levels of Occludin and ZO-1 genes in the DSS group were significantly decreased (*p* < 0.001) (Figure 4B,C). There were also significant differences between the SB, HSB, and BG groups compared to the control group, but the decreases in the expression levels of Occludin (*p* < 0.01) and ZO-1 (*p* < 0.05) genes were significantly lower than those in the DSS group. At the mRNA expression level, it is also indicated that *S. boulardii*, heat-killed *S. boulardii*, and *S. boulardii* β-glucan can restore the decreased expression levels of colonic tight junction proteins induced by DSS, suggesting their potential to protect intestinal barrier integrity.

### 3.4. Heat-Killed S. boulardii Decreased the Levels of Pro-Inflammatory Cytokines in the Serum and the Expression Levels of Pro-Inflammatory Cytokine mRNA in the Colon

**Figure 5:**
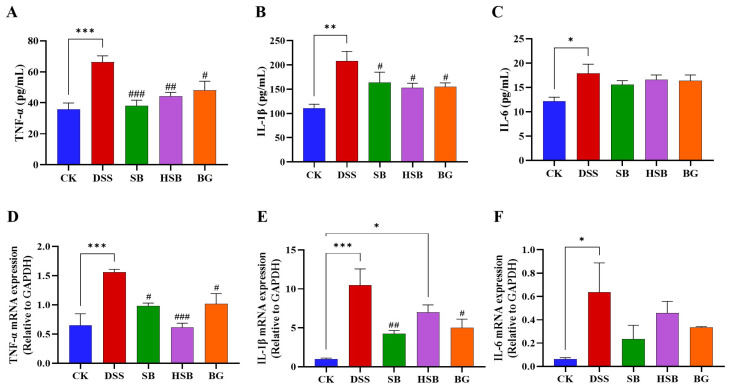
Effects of heat-killed *S. boulardii* on the levels of pro-inflammatory cytokines in serum and in the colon of mice with DSS-induced colitis. (**A**) The level of TNF-α in serum. (**B**) The level of IL-1β in serum. (**C**) The level of IL-6 in serum. (**D**) The mRNA expression level of TNF-α in the colon. (**E**) The mRNA expression level of IL-1β in the colon. (**F**) The mRNA expression level of IL-6 in the colon. Note: Data are expressed as mean ± SEM. A comparison of significant differences between groups is indicated, where * *p* < 0.05, ** *p* < 0.01, and *** *p* < 0.001 compared with the CK group; *# p* < 0.05, *## p* < 0.01, and *### p* < 0.001 compared with the DSS group.

Elevated expression levels of pro-inflammatory cytokine genes are also a typical feature of colitis and can be used to reflect the occurrence and severity of colonic inflammation. After measuring the levels of pro-inflammatory cytokines in the serum of each group of mice, we found that compared to the control group, the levels of TNF-α (*p* < 0.001), IL-1β (*p* < 0.01), and IL-6 (*p* < 0.05) were significantly increased in the serum of mice with DSS-induced colitis. When comparing the DSS group with the SB, HSB, and BG groups, all three substances significantly reduced the levels of TNF-α (DSS vs. SB, *p* < 0.001; DSS vs. HSB, *p* < 0.01; DSS vs. BG, *p* < 0.05) and IL-1β (*p* < 0.05) in mouse serum (Figure 5A,B). However, compared to the DSS group, although the levels of IL-6 in the serum of mice from each treatment group showed a decrease, it was not significant (*p* > 0.05) (Figure 5C).

By measuring the levels of pro-inflammatory cytokines in the mouse serum, we understand the severity of the inflammatory response and the release of pro-inflammatory cytokines. To further investigate the expression levels of pro-inflammatory cytokine mRNA in the colon and gain a more comprehensive understanding of the mechanisms underlying the inflammatory response, we utilized the RT-qPCR technique to determine the levels of pro-inflammatory cytokines in the colons of mice from different groups. Compared to the control group, the expression levels of the TNF-α (*p* < 0.001), IL-1β (*p* < 0.001), and IL-6 (*p* < 0.05) genes were significantly increased in the DSS-induced colitis group. Compared to the DSS group, the oral administration of *S. boulardii* and *β*-glucan significantly reduced the levels of TNF-α (DSS vs. SB, BG *p* < 0.05) and IL-1β (DSS vs. SB, *p* < 0.01; DSS vs. BG, *p* < 0.05) in the colon (Figure 5D,E). Similar to the changes in the IL-6 levels in the serum, the gene expression levels of IL-6 in the colon also showed no significant difference among the different treatment groups compared to that of the DSS-induced colitis mice (*p* > 0.05). (Figure 5F). It is worth noting that, compared to the intervention effects of *S. boulardii* and *β*-glucan, the intervention with heat-killed *S. boulardii* significantly reduced the expression level of TNF-α (*p* < 0.001), but did not significantly decrease the expression of IL-1β (*p >* 0.05).

Overall, *S. boulardii*, heat-killed *S. boulardii*, and *S. boulardii* β-glucan treatments all demonstrated the ability to reduce the levels of pro-inflammatory cytokines in the serum and decrease the expression levels of pro-inflammatory cytokine mRNA in the colon.

### 3.5. Heat-Killed S. boulardii Regulated the Gut Microbiota of DSS-Induced Colitis Mice

#### 3.5.1. Analysis of the Diversity and Overall Structure of the Gut Microbiota

**Figure 6:**
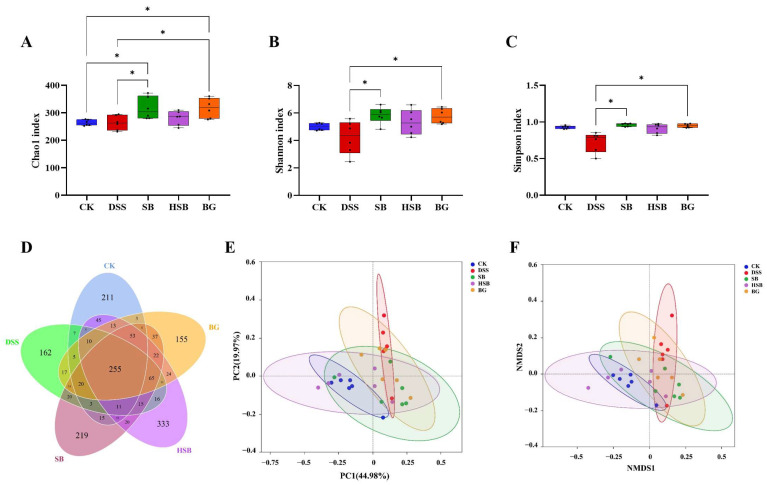
Effects of heat-killed *S. boulardii* on the diversity and overall structure of the gut microbiota. (**A**) Chao1 index. (**B**) Shannon index. (**C**) Simpson index. (**D**) Venn diagram. (**E**) PCoA. (**F**) NMDS analysis (Stress = 0.109). Note: Data are expressed as mean ± SEM. A comparison of significant differences between groups is indicated, where * *p* < 0.05.

Based on evaluations of the Chao1 index, Shannon index, and Simpson index, we assessed the α diversity of the gut microbiota in each group. Compared to the DSS group mice, both the *S. boulardii* and *S. boulardii* β-glucan treatments significantly increased all three α diversity indices (Figure 6A–C), indicating that *S. boulardii* and *S. boulardii* β-glucan can induce significant changes in the overall diversity of gut microbiota in DSS-induced colitis mice. Compared to the control group mice, probiotic and prebiotic treatment significantly increased the Chao1 index of gut microbiota in DSS-induced colitis mice (Figure 6A). It is worth noting that there were no significant differences between the heat-killed *S. boulardii* postbiotic treatment group and other groups, indicating that the intervention of heat-killed *S. boulardii* did not cause significant fluctuations in the overall diversity of the gut microbiota.

In this study, the impacts of three treatment methods on the gut microbiota of DSS-induced colitis mice were investigated by comparing the unique and shared OTU numbers among five groups. The control group, DSS group, SB group, HSB group, and BG group had 697, 655, 829, 933, and 729 OTUs, respectively. The numbers of unique OTUs were 211, 162, 219, 333, and 155, respectively (Figure 6D). Among them, the group treated with heat-killed *S. boulardii* showed the highest total number of OTUs as well as unique OTUs.

To further understand the differences in the overall structure of the gut microbiota among the groups, principal component analysis (PCoA) and non-metric multidimensional scaling (NMDS) analysis were performed to cluster the samples from different treatment groups. The results show a clear separation between the control group and the DSS group, and the three treatment groups showed varying degrees of proximity to the control group, with the HSB group being the closest (Figure 6E,F). These clustering situations indicate that *S. boulardii*, heat-killed *S. boulardii*, and *S. boulardii* β-glucan can restore the gut microbiota structure of DSS-induced colitis mice to a normal state to some extent, with the heat-killed *S. boulardii* showing the most effective restoration effect.

#### 3.5.2. Analysis of the Gut Microbiota Composition

The composition of the gut microbiota plays a crucial role in maintaining intestinal microbial homeostasis. To further understand the composition of gut microbiota in different groups of mice, we investigated the changes in bacterial abundance at the phylum and genus levels to assess the impact of *S. boulardii*, heat-killed *S. boulardii*, and *S. boulardii* β-glucan on the composition of gut microbiota in DSS-induced colitis mice. The CK group and DSS group represented the composition of gut microbiota in normal mice and DSS-induced colitis mice, respectively, which were used to accurately and intuitively determine the differences in community composition among the groups.

**Figure 7:**
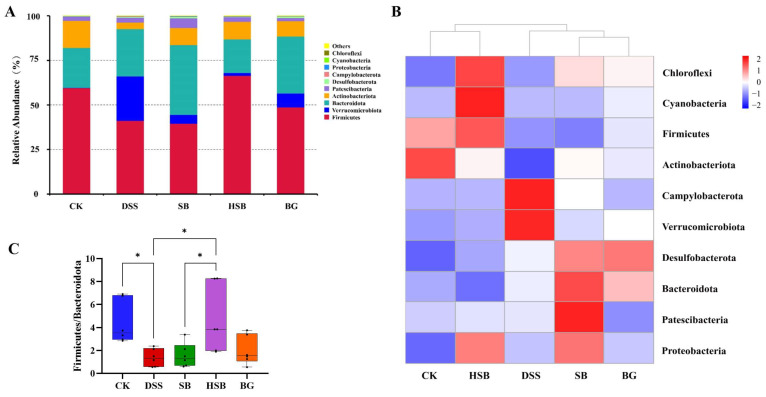
Effect of heat-killed *S. boulardii* on the composition of gut microbiota at the phylum level. (**A**) The relative abundance of gut microbiota at the phylum level. (**B**) Abundance clustering heatmap at the phylum level. (**C**) Firmicutes/Bacteroidota. Note: Data are expressed as mean ± SEM. A comparison of significant differences between groups is indicated, where * *p* < 0.05.

At the phylum level, Firmicutes, Verrucomicrobia, Bacteroidetes, and Actinobacteria were the most abundant microbial taxa in the mouse intestines, accounting for over 96% of the gut microbiota (Figure 7A). Compared to the control group, the relative abundance of Firmicutes decreased, while the relative abundance of Verrucomicrobia increased in the DSS group, SB group, and BG group. Among the treatment groups, the gut microbiota composition of the HSB group was closest to that of the CK group, as confirmed by the abundance clustering heatmap at the phylum level (Figure 7B). Additionally, compared to the control group, the ratios of Firmicutes/Bacteroidetes and Firmicutes/Bacteroidetes significantly decreased in the DSS group and SB group (*p* < 0.05), while the ratio of Firmicutes/Bacteroidetes showed a decreasing trend in the BG group but was not significant (*p >* 0.05). Only the HSB group significantly prevented the decrease in the Firmicutes/Bacteroidetes ratio in DSS-induced colitis mice (*p* < 0.05), further indicating the ability of heat-killed *S. boulardii* to restore gut microbial homeostasis in DSS-induced colitis mice (Figure 7C).

**Figure 8:**
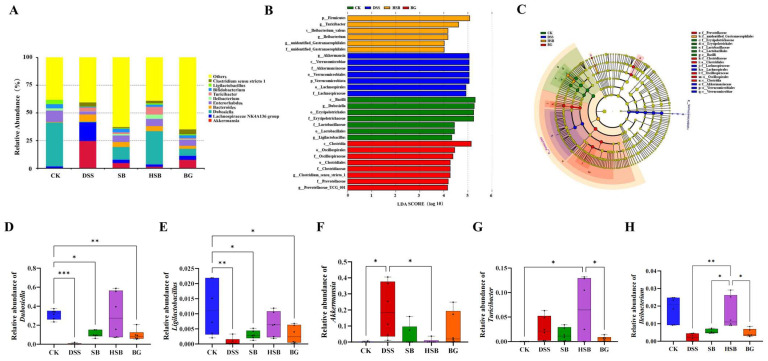
Effect of heat-killed *S. boulardii* on the composition of gut microbiota at the genus level. (**A**) The relative abundance of gut microbiota at the genus level. (**B**) Indicator bacteria with LDA scores of >4 in five groups. (**C**) LEfSe cladogram. (**D**) Comparison of relative abundance differences in *Dubosiella* between each group and the CK group. (**E**) Comparison of relative abundance differences in *Ligilactobacillus* between each group and the CK group. (**F**) Comparison of relative abundance differences in *Akkermansia* between each group and the DSS group. (**G**) Comparison of relative abundance differences in *Turicibacter* between each group and the HSB group. (**H**) Comparison of relative abundance differences in *Ileibacterium* between each group and the HSB group. Note: Data are expressed as mean ± SEM. A comparison of significant differences between groups is indicated, where * *p* < 0.05, ** *p* < 0.01, and *** *p* < 0.001.

At the genus level, we listed the top ten abundant species in horizontal abundance ranking, which are Akkermansia, Lachnospiraceae NK4A136 group, Dubosiella, Bacteroides, Enterorhabdus, Ileibacterium, Turicibacter, Bifidobacterium, Ligilactobacillus, and Clostridium sensu stricto 1 (Figure 8A). Compared to the control group, the DSS treatment led to significant changes in the composition of the mouse gut microbiota at the genus level.

To identify biomarkers for each group, we used linear discriminant analysis effect size (LEfSe) analysis to determine the differences in gut composition between groups. At the genus level, the dominant bacteria in the control group were *Dubosiella* and *Ligilactobacillus*, while *Akkermansia* was the dominant bacteria in the DSS group. *Turicibacter* and *Ileibacterium* were the dominant bacteria in the HSB group, and *Clostridium sensu stricto 1* and *Prevotellaceae UCG 001* were the dominant bacteria in the BG group. There were no significant differences in species between the BLD group and the other four groups (Figure 8B,C).

To further understand the effects of heat-killed *S. boulardii* on the gut microbiota of DSS-induced colitis mice, we performed differential analysis on the marker genera of the control, DSS, and HSB groups. The results show that there were significant differences (*p* < 0.05) in the marker genera *Dubosiella* and *Ligilactobacillus* between the control group and the DSS, SB, and BG groups, with the most significant difference observed between the DSS group and the control group (*Dubosiella p* < 0.001, *Ligilactobacillus p* < 0.01). The relative abundances of *Dubosiella* and *Ligilactobacillus* in the HSB group did not decrease significantly (*p >* 0.05) (Figure 8D,E). The relative abundance of *Akkermansia*, a marker genus in the DSS group, significantly decreased in the CK group and HSB group (*p* < 0.05) (Figure 8F). The marker genera in the HSB group were *Turicibacter* and *Ileibacterium*. Compared to the control group and BG group, the relative abundance of Turicibacter in the HSB group significantly increased (*p* < 0.05) (Figure 8G). Compared to the DSS, SB, and BG groups, the relative abundance of *Ileibacterium* in the HSB group significantly increased (*p* < 0.05), with the most significant difference observed between the DSS group and the HSB group (*p* < 0.01) (Figure 8H).

These results indicate that the heat-killed *S. boulardii* treatment can more effectively restore the decreases in *Dubosiella*, *Ligilactobacillus*, and *Ileibacterium* induced by DSS, and inhibit the increase in *Akkermansia* compared to live *S. boulardii* and *S. boulardii β*-glucan. This suggests that heat-killed *S. boulardii* can restore gut microbiota imbalance in DSS-induced colitis mice. It is worth noting that the heat-killed *S. boulardii* treatment significantly increases the abundance of *Turicibacter* in the mouse gut, whereas this genus is almost absent in the control group.

#### 3.5.3. Functional Prediction of the Gut Microbiota

**Figure 9:**
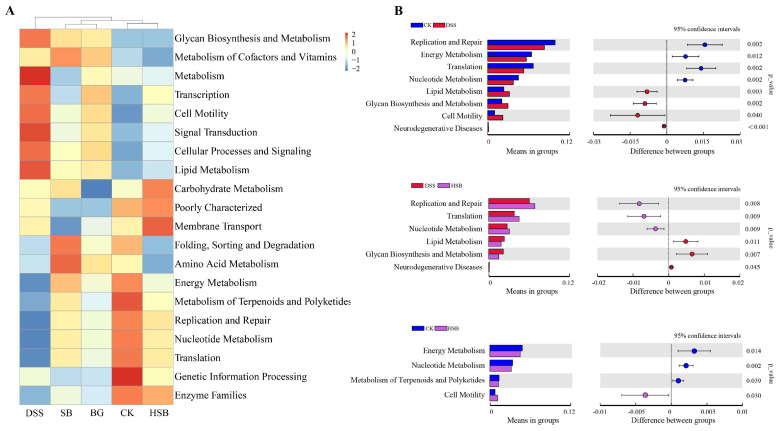
Prediction of the effect of heat-killed *S. boulardii* on the functional profile of the gut microbiota. (**A**) Heatmap of Pathway Level2. (**B**) Important pathways associated between two groups.

In addition to analyzing compositional differences at the phylum and genus levels, we used the PICRUSt algorithm to predict the macro-genomic profile of the small intestinal microbiota. In order to understand the impact of heat-killed *S. boulardii* on the gut microbial functional profile in DSS-induced colitis mice, we compared the significant differential metabolic pathways between the control group, DSS group, and HSB group. Overall, the intestinal microbiota was found to be mainly involved in various pathways such as Glycan Biosynthesis and Metabolism, Lipid Metabolism, Carbohydrate Metabolism, and Energy Metabolism (Figure 9A). Compared to the control group, the DSS-induced colitis mice showed a significant upregulation in Lipid Metabolism, Glycan Biosynthesis and Metabolism, Cell Motility, and Neurodegenerative Diseases pathways, while the Replication and Repair, Energy Metabolism, Translation, and Nucleotide Metabolism pathways were significantly downregulated (*p* < 0.05). Compared to the DSS group, treatment with heat-killed *S. boulardii* significantly upregulated the Replication and Repair, Translation, and Nucleotide Metabolism pathways, and downregulated the Lipid Metabolism, Glycan Biosynthesis and Metabolism, and Neurodegenerative Disease pathways in DSS-induced colitis mice (*p* < 0.05). This indicates that heat-killed *S. boulardii* can effectively restore abnormalities in certain metabolic pathways induced by DSS. Compared to the control group, the HSB group showed a significant upregulation in the Cell Motility pathway and downregulation in the Energy Metabolism, Nucleotide Metabolism, and Metabolism of Terpenoids and Polyketides pathways (*p* < 0.05) (Figure 9B).

## 4. Discussion

UC is a chronic and recurrent disease that is difficult to cure. Individuals with a history of UC are considered to be at high risk for developing colorectal cancer [18,19]. The main goals of UC treatment are achieving clinical remission, preventing complications, and improving patients’ quality of life [20]. Although conventional medications such as aminosalicylates and immunosuppressive agents can provide relief for UC, they often have poor sustainability, high cost, and a tendency for relapse, causing significant physical and psychological distress for patients [21,22,23]. Therefore, finding effective and cost-efficient novel biological therapeutics is highly meaningful [24]. In this study, we investigated the effects of three beneficial substances for the gut, *S. boulardii*, heat-killed *S. boulardii*, and *S. boulardii* β-glucan, on DSS-induced mouse colitis. By comparing the effects of these three substances, we highlighted the potential of heat-killed *S. boulardii* postbiotics in treating UC. The results of the study show that *S. boulardii*, heat-killed *S. boulardii*, and *S. boulardii* β-glucan all alleviated colitis symptoms, restored intestinal barrier damage, and modulated gut microbiota. Among them, heat-killed *S. boulardii* demonstrated a significant advantage in restoring the dysbiosis of the gut microbiota in DSS-induced colitis mice.

The most typical clinical manifestations of UC are bloody stools, diarrhea, and weight loss [25,26]. In this study, using a DSS-induced mouse model of UC, we found that treatments with *S. boulardii*, heat-killed *S. boulardii*, and *S. boulardii* β-glucan significantly alleviated the clinical symptoms of DSS-induced colitis mice. This was evidenced by the recovery of body weight and a decrease in the DAI. Organ indices can reflect the physiological status of key organs under diseased conditions. In particular, the spleen index is closely related to adaptive immune responses as the spleen serves as a site for immune cell proliferation and differentiation [27]. Previous studies have shown that the spleen enlarges under immune activation during the occurrence and development of colitis [28,29]. We observed that DSS induction significantly increased the spleen index in mice, while treatment with *S. boulardii*, heat-killed *S. boulardii*, and *S. boulardii* β-glucan reduced the spleen index, indicating an immunomodulatory effect on UC mice.

Studies on UC have consistently shown that DSS induction leads to significant changes in the colonic morphology and epithelial structure [30,31]. Our research demonstrated that treatments with *S. boulardii*, heat-killed *S. boulardii*, and S. *boulardii* β-glucan can inhibit colon shortening, significantly improve the structure and function of the intestine, and restore its protective barrier function by reducing crypt loss, intestinal villi damage, and inflammatory cell infiltration caused by DSS stimulation. The expanded intestinal inflammation and associated epithelial barrier dysfunction are closely related in UC [6]. The integrity of the intestinal epithelial barrier structure and function is regulated by tight junction proteins Occludin and ZO-1, and the levels of epithelial Occludin and ZO-1 can reflect the status of barrier damage [32]. *S. boulardii*, heat-killed *S. boulardii*, and *S. boulardii β*-glucan upregulated the levels of tight junction proteins Occludin and ZO-1, indicating that they can also restore the structure and function of the epithelial barrier by increasing the levels of tight junction proteins. Additionally, all three treatment substances can reduce the expression of pro-inflammatory cytokines TNF-α, IL-1β, and IL-6 in both serum and the colon. Notably, the use of heat-killed *S. boulardii* enhanced the inhibitory effect on the expression of TNF-α mRNA in the colon.

The gut microbiota plays a crucial role in maintaining host health [33]. Patients with common gastrointestinal disorders, such as IBD and irritable bowel syndrome (IBS), exhibit differences in gut microbiota composition and functionality compared to healthy individuals [34]. In this study, we observed that heat-killed *S. boulardii* showed a superior regulation of the gut microbiota compared to *S. boulardii* and *S. boulardii* β-glucan. Firstly, heat-killed *S. boulardii* significantly increased the diversity of the gut microbiota in DSS-induced colitis mice. Secondly, cluster analysis revealed that heat-killed *S. boulardii* was most effective in restoring the overall structure of the gut microbiota to a normal state compared to the other treatment groups. Importantly, intervention with heat-killed *S. boulardii* did not affect the α diversity of the gut microbiota, providing favorable evidence for the safety of postbiotic interventions. Additionally, we analyzed the impact of *S. boulardii*, heat-killed *S. boulardii*, and *S. boulardii* β-glucan on the microbial composition at the phylum and genus levels in DSS-induced colitis mice. At the phylum level, the decrease in the Firmicutes/Bacteroidetes ratio is associated with inflammation [35,36], Our research results demonstrate a significant decrease in the Firmicutes/Bacteroidetes ratio induced by DSS, which can be inhibited only through treatment with heat-killed *S. boulardii*. To further understand the mechanism by which heat-killed *S. boulardii* regulates the gut microbiota, we focused on analyzing its impact on signature bacterial genera in DSS-induced colitis mice. Previous studies have shown that interventions with beneficial substances can reshape the gut microbiota in colitis mice, leading to significant changes in certain bacterial genera [37,38]. In our study, we observed that compared to DSS-induced model mice, treatment with heat-killed *S. boulardii* increased the abundance of beneficial bacterial genera, including *Dubosiella* [39], *Ligilactobacillus* [40,41], *Turicibacter* [42,43], and *Ileibacterium* [44]. The growths of these genera were significantly suppressed in DSS model mice, indicating that heat-killed *S. boulardii* intervention can regulate the gut microbiota by restoring the abundance of dominant bacteria, thus achieving a new balance. It is worth noting that individual studies have suggested that some Turicibacter bacteria may exhibit pathogenic characteristics, reducing butyrate levels in the intestine and often being associated with host inflammation [45,46]. This also indicates that there are uncertainties and unknown factors in the promotion of gut health by postbiotics, highlighting the need for further in-depth research. Lastly, through PICRUSt analysis, we found that heat-killed *S. boulardii* can partially restore the abnormal metabolic pathways caused by DSS. This research provides evidence that postbiotics can improve immune-related diseases such as intestinal inflammation by modulating the functional capacity of the gut microbiota.

It should be noted that although DSS-induced colitis is widely used as an animal model for UC, and in this study, although we observed some typical pathological changes similar to human UC in DSS-induced colitis mice, this does not fully reflect the complexity of human UC. Therefore, our study merely demonstrated the potential of *S. boulardii*, heat-killed *S. boulardii*, and *S. boulardii* β-glucan in treating human UC based on the mouse UC model. To determine whether *S. boulardii*, heat-killed *S. boulardii*, and *S. boulardii* β-glucan can truly be used in the actual treatment of human UC, further validation with clinical observations and other animal models is needed in future research.

## 5. Conclusions

In summary, heat-killed *S. boulardii* can alleviate DSS-induced ulcerative colitis in mice by restoring the intestinal barrier, reducing inflammation, and modulating the gut microbiota. In particular, in terms of gut microbiota modulation, postbiotics may have more significant therapeutic effects compared to probiotics and prebiotics. Our research highlights the potential of postbiotics as a promising novel biological therapy for treating ulcerative colitis.

## Data Availability

Data are contained within the article.

## Supplementary Materials

The following supporting information can be downloaded at https://www.mdpi.com/article/10.3390/nu16050702/s1: Table S1: Disease activity index scoring criteria; Table S2: Sequences of primers used for quantitative real-time PCR (RT-qPCR). Figure S1. Identification of *S. boulardii*. (A) Colony morphology on YPD agar. (B) Cell morphology under a light microscope after staining with methylene blue (1000×). (C) Phylogenetic tree constructed from the alignment and comparison of 16S rRNA gene sequences using NCBI (X-1 represents the *S. boulardii* strain selected in this study).

## References

[1] Molodecky N.A., Soon I.S., Rabi D.M., Ghali W.A., Ferris M., Chernoff G., Benchimol E.I., Panaccione R., Ghosh S., Barkema H.W. (2012). Increasing Incidence and Prevalence of the Inflammatory Bowel Diseases with Time, Based on Systematic Review. Gastroenterology.

[2] Burisch J., Munkholm P. (2015). The Epidemiology of Inflammatory Bowel Disease. Scand. J. Gastroenterol..

[3] Frolkis A.D., Dykeman J., Negrón M.E., Debruyn J., Jette N., Fiest K.M., Frolkis T., Barkema H.W., Rioux K.P., Panaccione R. (2013). Risk of Surgery for Inflammatory Bowel Diseases Has Decreased over Time: A Systematic Review and Meta-Analysis of Population-Based Studies. Gastroenterology.

[4] Ungaro R., Mehandru S., Allen P.B., Peyrin-Biroulet L., Colombel J.-F. (2017). Ulcerative Colitis. Lancet.

[5] Hvas C.L., Bendix M., Dige A., Dahlerup J.F., Agnholt J. (2018). Current, Experimental, and Future Treatments in Inflammatory Bowel Disease: A Clinical Review. Immunopharmacol. Immunotoxicol..

[6] Le Berre C., Honap S., Peyrin-Biroulet L. (2023). Ulcerative Colitis. Lancet.

[7] Asquith M., Powrie F. (2010). An Innately Dangerous Balancing Act: Intestinal Homeostasis, Inflammation, and Colitis-Associated Cancer. J. Exp. Med..

[8] Chan M.Z.A., Liu S.-Q. (2022). Fortifying Foods with Synbiotic and Postbiotic Preparations of the Probiotic Yeast, Saccharomyces Boulardii. Curr. Opin. Food Sci..

[9] Sivananthan K., Petersen A.M. (2018). Review of Saccharomyces Boulardii as a Treatment Option in IBD. Immunopharmacol. Immunotoxicol..

[10] Li B., Zhang H., Shi L., Li R., Luo Y., Deng Y., Li S., Li R., Liu Z. (2022). *Saccharomyces Boulardii* Alleviates DSS-Induced Intestinal Barrier Dysfunction and Inflammation in Humanized Mice. Food Funct..

[11] Collado M.C., Vinderola G., Salminen S. (2019). Postbiotics: Facts and Open Questions. A Position Paper on the Need for a Consensus Definition. Benef. Microbes.

[12] Lavelle A., Sokol H. (2020). Gut Microbiota-Derived Metabolites as Key Actors in Inflammatory Bowel Disease. Nat. Rev. Gastroenterol. Hepatol..

[13] Aguilar-Toalá J.E., Garcia-Varela R., Garcia H.S., Mata-Haro V., González-Córdova A.F., Vallejo-Cordoba B., Hernández-Mendoza A. (2018). Postbiotics: An Evolving Term within the Functional Foods Field. Trends Food Sci. Technol..

[14] Salminen S., Collado M.C., Endo A., Hill C., Lebeer S., Quigley E.M.M., Sanders M.E., Shamir R., Swann J.R., Szajewska H. (2021). The International Scientific Association of Probiotics and Prebiotics (ISAPP) Consensus Statement on the Definition and Scope of Postbiotics. Nat. Rev. Gastroenterol. Hepatol..

[15] Han F., Fan H., Yao M., Yang S., Han J. (2017). Oral Administration of Yeast β-Glucan Ameliorates Inflammation and Intestinal Barrier in Dextran Sodium Sulfate-Induced Acute Colitis. J. Funct. Foods.

[16] Jawhara S., Habib K., Maggiotto F., Pignede G., Vandekerckove P., Maes E., Dubuquoy L., Fontaine T., Guerardel Y., Poulain D. (2012). Modulation of Intestinal Inflammation by Yeasts and Cell Wall Extracts: Strain Dependence and Unexpected Anti-Inflammatory Role of Glucan Fractions. PLoS ONE.

[17] Liu Y., Wu Q., Wu X., Algharib S.A., Gong F., Hu J., Luo W., Zhou M., Pan Y., Yan Y. (2021). Structure, Preparation, Modification, and Bioactivities of β-Glucan and Mannan from Yeast Cell Wall: A Review. Int. J. Biol. Macromol..

[18] Dai N., Haidar O., Askari A., Segal J.P. (2023). Colectomy Rates in Ulcerative Colitis: A Systematic Review and Meta-Analysis. Dig. Liver Dis..

[19] Zhang M., Lv H., Yang H., Zhang H., Bai X., Qian J. (2023). Elderly Patients with Moderate-To-Severe Ulcerative Colitis Are More Likely to Have Treatment Failure and Adverse Outcome. Gerontology.

[20] Ferretti F., Cannatelli R., Monico M.C., Maconi G., Ardizzone S. (2022). An Update on Current Pharmacotherapeutic Options for the Treatment of Ulcerative Colitis. J. Clin. Med..

[21] Chapman T.P., Frias Gomes C., Louis E., Colombel J.-F., Satsangi J. (2020). Review Article: Withdrawal of 5-Aminosalicylates in Inflammatory Bowel Disease. Aliment. Pharmacol. Ther..

[22] Iborra M., Herreras J., Boscá-Watts M.M., Cortés X., Trejo G., Cerrillo E., Hervás D., Mínguez M., Beltrán B., Nos P. (2019). Withdrawal of Azathioprine in Inflammatory Bowel Disease Patients Who Sustain Remission: New Risk Factors for Relapse. Dig. Dis. Sci..

[23] Balram B., Lubov J., Theoret Y., Afif W., Bitton A., Wild G., Lakatos P.L., Bessissow T. (2021). Poor Drug Sustainability in Inflammatory Bowel Disease Patients in Clinical Remission on Thiopurine Monotherapy. Dig. Dis. Sci..

[24] Ma C., Guizzetti L., Cipriano L.E., Parker C.E., Nguyen T.M., Gregor J.C., Chande N., Feagan B.G., Jairath V. (2019). Systematic Review with Meta-Analysis: High Prevalence and Cost of Continued Aminosalicylate Use in Patients with Ulcerative Colitis Escalated to Immunosuppressive and Biological Therapies. Aliment. Pharmacol. Ther..

[25] Louis E., Van Kemseke C., Reenaers C. (2011). Necessity of Phenotypic Classification of Inflammatory Bowel Disease. Best. Pract. Res. Clin. Gastroenterol..

[26] Rubin D.T., Ananthakrishnan A.N., Siegel C.A., Sauer B.G., Long M.D. (2019). ACG Clinical Guideline: Ulcerative Colitis in Adults. Am. J. Gastroenterol..

[27] Zhang Q., Xiang L., Zaman M.H., Dong W., He G., Deng G.-M. (2020). Predominant Role of Immunoglobulin G in the Pathogenesis of Splenomegaly in Murine Lupus. Front. Immunol..

[28] Aquila G., Re Cecconi A.D., Forti M., Frapolli R., Bello E., Novelli D., Russo I., Licandro S.A., Staszewsky L., Martinelli G.B. (2020). Trabectedin and Lurbinectedin Extend Survival of Mice Bearing C26 Colon Adenocarcinoma, without Affecting Tumor Growth or Cachexia. Cancers.

[29] Tsai W.-C., Wong W.-T., Hsu H.-T., Cheng Y.-H., Yu Y.-H., Chen W.-J., Ho C.-L., Hsu H.-C., Hua K.-F. (2022). Surfactin Containing Bacillus Licheniformis-Fermented Products Alleviate Dextran Sulfate Sodium-Induced Colitis by Inhibiting Colonic Inflammation and the NLRP3 Inflammasome in Mice. Animals.

[30] Jiang S., Xu H., Zhao C., Zhong F., Li D. (2023). Oyster Polysaccharides Relieve DSS-Induced Colitis via Anti-Inflammatory and Maintaining the Physiological Hypoxia. Int. J. Biol. Macromol..

[31] Xu D., Wu Q., Liu W., Hu G., Meng H., Wang J. (2023). Therapeutic Efficacy and Underlying Mechanisms of Gastrodia Elata Polysaccharides on Dextran Sulfate Sodium-Induced Inflammatory Bowel Disease in Mice: Modulation of the Gut Microbiota and Improvement of Metabolic Disorders. Int. J. Biol. Macromol..

[32] Hall C.H.T., Lee J.S., Murphy E.M., Gerich M.E., Dran R., Glover L.E., Abdulla Z.I., Skelton M.R., Colgan S.P. (2020). Creatine Transporter, Reduced in Colon Tissues from Patients with Inflammatory Bowel Diseases, Regulates Energy Balance in Intestinal Epithelial Cells, Epithelial Integrity, and Barrier Function. Gastroenterology.

[33] Round J.L., Mazmanian S.K. (2009). The Gut Microbiota Shapes Intestinal Immune Responses during Health and Disease. Nat. Rev. Immunol..

[34] Lee T., Clavel T., Smirnov K., Schmidt A., Lagkouvardos I., Walker A., Lucio M., Michalke B., Schmitt-Kopplin P., Fedorak R. (2017). Oral versus Intravenous Iron Replacement Therapy Distinctly Alters the Gut Microbiota and Metabolome in Patients with IBD. Gut.

[35] Stojanov S., Berlec A., Štrukelj B. (2020). The Influence of Probiotics on the Firmicutes/Bacteroidetes Ratio in the Treatment of Obesity and Inflammatory Bowel Disease. Microorganisms.

[36] Hou K., Wu Z.-X., Chen X.-Y., Wang J.-Q., Zhang D., Xiao C., Zhu D., Koya J.B., Wei L., Li J. (2022). Microbiota in Health and Diseases. Signal Transduct. Target. Ther..

[37] He X., Liu J., Long G., Xia X.-H., Liu M. (2021). 2,3,5,4′-Tetrahydroxystilbene-2-O-β-D-Glucoside, a Major Bioactive Component from Polygoni Multiflori Radix (Heshouwu) Suppresses DSS Induced Acute Colitis in BALb/c Mice by Modulating Gut Microbiota. Biomed. Pharmacother..

[38] Feng C., Zhang W., Zhang T., He Q., Kwok L.-Y., Tan Y., Zhang H. (2022). Heat-Killed Bifidobacterium Bifidum B1628 May Alleviate Dextran Sulfate Sodium-Induced Colitis in Mice, and the Anti-Inflammatory Effect Is Associated with Gut Microbiota Modulation. Nutrients.

[39] Liu T.-H., Wang J., Zhang C.-Y., Zhao L., Sheng Y.-Y., Tao G.-S., Xue Y.-Z. (2023). Gut Microbial Characteristical Comparison Reveals Potential Anti-Aging Function of Dubosiella Newyorkensis in Mice. Front. Endocrinol..

[40] Abramov V.M., Kosarev I.V., Machulin A.V., Deryusheva E.I., Priputnevich T.V., Panin A.N., Chikileva I.O., Abashina T.N., Manoyan A.M., Ahmetzyanova A.A. (2023). Ligilactobacillus Salivarius 7247 Strain: Probiotic Properties and Anti-Salmonella Effect with Prebiotics. Antibiotics.

[41] Guerrero Sanchez M., Passot S., Campoy S., Olivares M., Fonseca F. (2022). Ligilactobacillus Salivarius Functionalities, Applications, and Manufacturing Challenges. Appl. Microbiol. Biotechnol..

[42] Wang E., Zhou Y., Liang Y., Ling F., Xue X., He X., Zhai X., Xue Y., Zhou C., Tang G. (2022). Rice Flowering Improves the Muscle Nutrient, Intestinal Microbiota Diversity, and Liver Metabolism Profiles of Tilapia (Oreochromis Niloticus) in Rice-Fish Symbiosis. Microbiome.

[43] Lynch J.B., Gonzalez E.L., Choy K., Faull K.F., Jewell T., Arellano A., Liang J., Yu K.B., Paramo J., Hsiao E.Y. (2023). Gut Microbiota Turicibacter Strains Differentially Modify Bile Acids and Host Lipids. Nat. Commun..

[44] Wang Y., Ablimit N., Zhang Y., Li J., Wang X., Liu J., Miao T., Wu L., Wang H., Wang Z. (2021). Novel β-Mannanase/GLP-1 Fusion Peptide High Effectively Ameliorates Obesity in a Mouse Model by Modifying Balance of Gut Microbiota. Int. J. Biol. Macromol..

[45] Fung T.C., Vuong H.E., Luna C.D.G., Pronovost G.N., Aleksandrova A.A., Riley N.G., Vavilina A., McGinn J., Rendon T., Forrest L.R. (2019). Intestinal Serotonin and Fluoxetine Exposure Modulate Bacterial Colonization in the Gut. Nat. Microbiol..

[46] Chung Y., Ryu Y., An B.C., Yoon Y.-S., Choi O., Kim T.Y., Yoon J., Ahn J.Y., Park H.J., Kwon S.-K. (2021). A Synthetic Probiotic Engineered for Colorectal Cancer Therapy Modulates Gut Microbiota. Microbiome.

