# Supplementary material for: Heat-Killed Saccharomyces boulardii Alleviates Dextran Sulfate Sodium-Induced Ulcerative Colitis by Restoring the Intestinal Barrier, Reducing Inflammation, and Modulating the Gut Microbiota
Source: Nutrients. 2024 Feb 29;16(5):702. doi: 10.3390/nu16050702 (PMC10934832; doi:10.3390/nu16050702)
Supplement: Supplementary file 1 [file nutrients-16-00702-s001.zip › nutrients-2885345-supplementary.pdf]

Supplementary

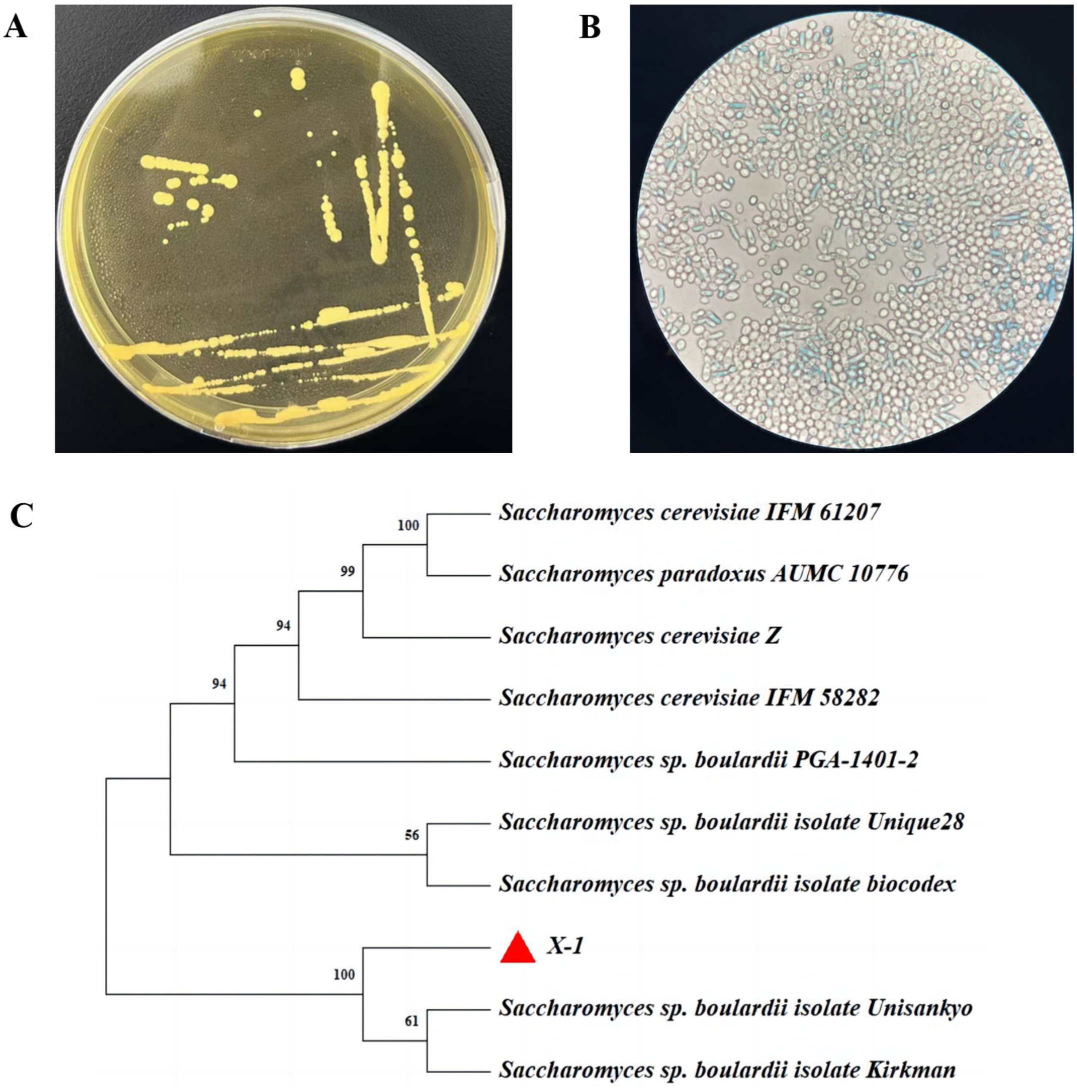

Figure S1. Identification of *S. boulardii*. (A) Colony morphology on YPD agar. (B) Cell morphology under a light microscope after staining with methylene blue (1000×). (C) Phylogenetic tree constructed from the alignment and comparison of 16S rRNA gene sequences using NCBI (X-1 represents the *S. boulardii* strain selected in this study).

Table S1. Disease activity index scoring criteria.

Score	Body weight loss（%）	Fecal traits	Hematochezia
0	≤0	Normal stools	Normal
1	1-5	Soft stools	Small amounts of blood-streaked feces
2	6-10	Loose stools	A certain amount of bloody feces
3	11-15	Semi-loose stools	Conspicuous blood-wrapped feces
4	＞15	Watery stools	Visible rectal bleeding

Table S2. Sequences of primers used for quantitative real-time PCR (RT-qPCR).

Target Gene	Nucleotide Sequence of Primer (5′ to 3′) Forward	Nucleotide Sequence of Primer (5′ to 3′) Reverse
TNF-α	CCCTCACACTCACAAACCAC	ACAAGGTACAACCCATCGGC
IL-1β	GTCGCTCAGGGTCACAAGAA	CCACACGTTGACAGCTAGGT
IL-6	GGAGCCCACCAAGAACGATA	GTCACCAGCATCAGTCCCAA
Occludin	TTTCCTGCGGTGACTTCTCC	AAAACAGTGGTGGGGAACGT
ZO-1	GAGCAGGCTTTGGAGGAGAC	TGGGACAAAAGTCCGGGAAG
GAPDH	ATGGTGAAGGTCGGTGTGAA	TTTGCCGTGAGTGGAGTCAT
